# Paradoxical induction of growth arrest and apoptosis by EGF via the up-regulation of PTEN by activating Redox factor-1/Egr-1 in human lung cancer cells

**DOI:** 10.18632/oncotarget.13809

**Published:** 2016-12-07

**Authors:** Je-won Ryu, Sung Sik Choe, Seung-Hee Ryu, Eun-Young Park, Byoung Wook Lee, Tae Keun Kim, Chang Hoon Ha, Sang-wook Lee

**Affiliations:** ^1^ Department of Radiation Oncology, Asan Medical Center and University of Ulsan College of Medicine, Seoul, Republic of Korea; ^2^ Department of Biological Sciences, Institute of Molecular Biology and Genetics, National Creative Research Institutive Center for Adipose Tissue Remodeling, Seoul National University, Seoul, Republic of Korea; ^3^ Asan Institute for Life Science, Asan Medical Center and University of Ulsan College of Medicine, Seoul, Republic of Korea; ^4^ Department of Life Science, College of Natural Science, Hallym University, Kyeongki Province, Republic of Korea

**Keywords:** Epidermal Growth Factor (EGF), Purinergic Receptor 2(P2Y), Redox Factor-1(Ref-1), Zinc Finger-containing Transcriptional Regulator 1(EGR1), Phosphatase and Tensin Homolog (PTEN)

## Abstract

Epidermal growth factor (EGF) signaling promotes cell proliferation and survival in several types of cancer. Here, however, we showed that EGF inhibits proliferation and promotes programmed cell death in non-small cell lung cancer (NSCLC) cells. In A549 cells, EGF increased redox factor-1 (Ref-1) expression and the association of Ref-1 with zinc finger-containing transcriptional regulator (EGR1) via activation of p22^phox^, RAC1, and an NADPH oxidase subunit. EGF increased p22^phox^ and RAC1 expression through activation of purinergic receptors (P2Y). Elevated Ref-1/EGR1 levels increased phosphatase and tensin homolog (PTEN) levels, leading to inhibition of the Akt pathway. EGF-induced PTEN upregulation increased apoptosis and autophagy-induced damage in A549 cells, whereas Ref-1 knockdown blocked EGF-induced PTEN upregulation in an NADPH oxidase p22^phox^ subunit-independent manner. In addition, p22^phox^ knockdown restored EGF-induced effects, implying that changes in P2Y activity caused by EGF, which activates NADPH oxidase via RAC1, influenced Ref-1-mediated redox regulation. Finally, EGF similarly attenuated cell proliferation and promoted autophagy and apoptosis *in vivo* in a xenograft model using A549 cells. These findings reveal that EGF-induced redox signaling is linked to Ref-1-induced death in NSCLC cells.

## INTRODUCTION

Inhibition of epidermal growth factor receptor (EGFR) signaling has been suggested as a therapeutic strategy for several types of cancer with high mortality rates, including non-small cell lung cancer (NSCLC) [1, 2]. The phosphoinositide 3-kinase/protein kinase B/mammalian target of rapamycin (PI3K/Akt/mTOR) pathway, which is downstream of EGFR signaling, may also play an important role in cancer. Moreover, loss of tumor suppressor phosphatase and tensin homolog (PTEN) expression results in hyperactivation of Akt signaling and dysregulated cell growth in NSCLC and other types of cancer [2–5]. Therefore, potential cancer treatments that modulate EGFR pathway activity have been extensively studied. Although EGFR signaling plays a key role in proliferation and differentiation, binding of the ligand EGF to EGFR1 and EGFR2 can induce programmed cell death [6–9]. Inhibition of EGFR-dependent survival signals promotes apoptotic signals, which synergistically increase tumor cell death [10]. Our previous studies suggest that exogenous administration of EGF inhibits growth and induces death in tumor cells [6, 11]. However, the mechanism by which EGF-EFGR signaling induces tumor cell death has not yet been fully described.

EGF-EGFR signaling activates of NADPH oxidase via RAC1, resulting in the production of reactive oxygen species (ROS) [10, 12, 13]. Specific genes likely promote adaptation to oxidative stress and regulate redox reaction. Notably, as purinergic receptors (P2Y) are G-coupled protein receptors, have eight subtypes (P2Y_1_, P2Y_2_, P2Y_4_, P2Y_6_, P2Y_11_, P2Y_12_, P2Y_13_, and P2Y_14_). All types of tumors express Purinergic receptors. Many studies have evaluated the potential applications of purinergic receptor targeting in cancer therapy. For example, five P2 receptor subtypes have been implicated in the inhibition of cancer cell growth, namely P2×5, P2×7, P2Y_1_, P2Y_2_, and P2Y_11_. In particular, P2Y_1_ receptors decrease cell proliferation in cancer cells. The P2Y_1_ receptor may play role in induction of cancer cell death or growth inhibition [14–17]. Binding extracellular ATP to P2Y, induces the accumulation of intracellular Ca^2+^ and ROS through RAC1-mediated NADPH oxidase activation [18]. Cells release extracellular ATP in response to stress, and this accumulation of ATP together with other stimuli can activate several growth factor receptors and transcriptional factors, such as EGR1[17]. Especially, in human lung cancer cells, release of extracellular ATP activates P2 receptors involved in autocrine signaling [19, 20]. In addition to EGFR mutations, PI3K/Akt survival signaling, PTEN-induced cell death, and other recently described mechanisms, continued study of EGF–EGFR signaling under conditions of oxidative stress might reveal changes in target gene activation. Such studies might help to establish EGF–EGFR-target genes-related therapies for NSCLC patients.

Redox factor-1 (Ref-1) is a multifunctional protein that contributes to both DNA repair and transcriptional regulation [21–23]. Notably, Ref-1 is highly expressed in cancer cells and can have post-translational modifications, protein-protein interactions, redox activity, and roles in DNA repair, suggesting that it could be a useful therapeutic target gene [21–23]. Ref-1 increases gene expression by directly associating with transcription factors, including AP-1, EGR1, NF-κB, p53, STAT3, and HIF1α. These associations can promote either cell survival or death by altering the activity of different subsets of cell fate-determining factors. Additionally, subcellular localization of Ref-1 may be associated with prognosis in cancer patients [23–25]. In response to oxidative stress, Ref-1 associates with EGR1 in the nucleus to increase PTEN activation, which inhibits cancer growth and ultimately leads to apoptosis [26–28].

We previously reported that EGF treatment reduces radiation-induced damage, including oral mucositis, radio-dermatitis, ulcers, and intestinal mucosal damage in cancer patients and rodent models to improve healing [29–34]. However, whether EGF increases growth in cancer tissues during after radiotherapy remains unknown. We therefore assessed whether EGF increased proliferation and survival in normal and cancer cells after radiation treatments and found that EGF induced cell death in EGFR1-overexpressing tumor cells [6]. Here, we found that activation of the Ref-1/EGR1 complex increased PTEN expression, in turn inhibiting the Akt pathway and the response to EGF-EGFR-mediated oxidative stress, in an NSCLC cell line.

## RESULTS

### EGF attenuates cell growth and colony formation in NSCLC cells

**Figure 1:**
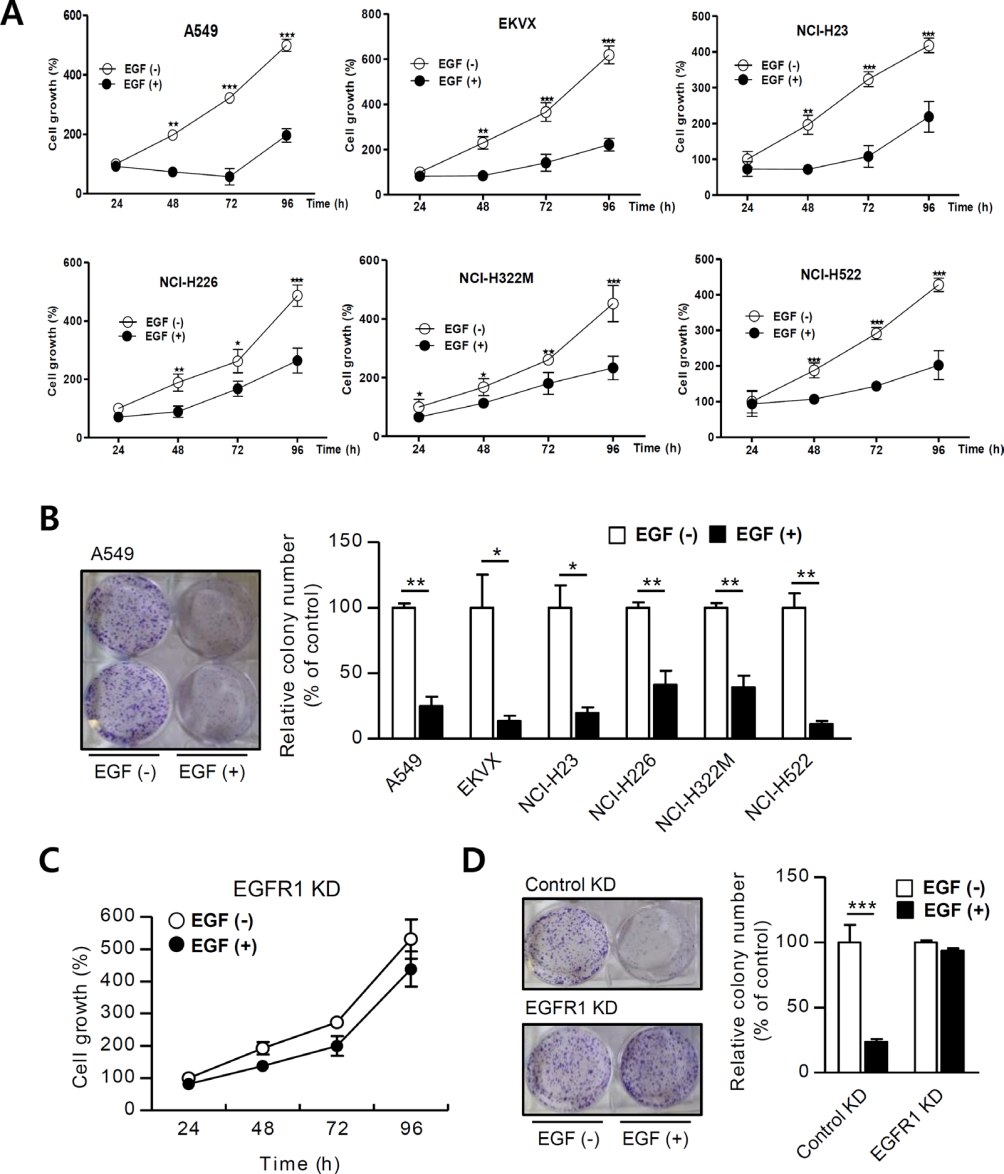
EGF inhibits NSCLC cell growth (**A**) NSCLC cells were incubated with EGF (100 ng/mL) and proliferation was assessed using MTT assays (*n* = 8). (**B**) Cells were treated with EGF once every 3 days for 15 days (*n* = 3), and changes in cell growth were quantitatively analyzed by counting colonies. EGFR1 KD cell growth was analyzed using (**C**) MTT assays and (**D**) colony counting. Means ± SDs of 3–8 wells are shown. Growth percent's are presented in the graph. Data are representative of three independent experiments and were analyzed using unpaired *t*-tests (*P < 0.05, **P < 0.01, ***P < 0.001).

EGFR1 and EGFR2 activate programmed cell death, which occurs after 24 to 48 h [7, 35]. We used MTT assays to assess whether EGF affects NSCLC cell growth. Growth rates decreased in A549 cells beginning 48 h after EGF treatment compared to control cells (at 48 h, 74 ± 6% vs. 198 ± 7%; at 72 h, 58 ± 28% vs. 322 ± 24%; and at 96 h, 196 ± 23% vs. 499 ± 20% in EGF-treated vs. control cells, respectively; Figure 1A Results obtained using other NSCLC cell lines (EKVX, H23, H322M, and H226 cells; Supplementary Table S1) were similar to those observed in A549 cells (Figure 1A). Clonogenic assays were then performed to confirm that chronic EGF treatment stimulates growth in NSCLC cells. Colony formation decreased in EGF-treated A549 cells compared to PBS-treated control cells (based on images of A549 colons; Figure 1B). Control cells grew approximately 75% faster than EGF-treated cells (100% ± 3% vs. 25% ± 7%, respectively; Figure 1B). The results obtained from colony formation assays in five other NSCLC cells were similar to those obtained using A549 cells (Figure 1B and based on colon images of six NSCLC cells; Supplementary Figure S1). These findings suggest that constitutive EGF activation inhibits growth in these NSCLC cells. We then generated a stable EGFR1 knock-down (KD) cell line, in which EGFR1 mRNA and protein levels were negligible, to investigate whether EGFR1 required for EGF-induced reductions in NSCLC growth (Supplementary Figure S2). EGF treatment did not affect growth rates and colony formation in EGFR1 KD cells (Figure 1C and 1D). Together, these results suggest that EGF-induced growth inhibition is EGFR-dependent and requires more than 24 h to take effect.

### EGF increases PTEN levels through ROS-induced Ref-1 and EGR1 expression in A549 cells

**Figure 2:**
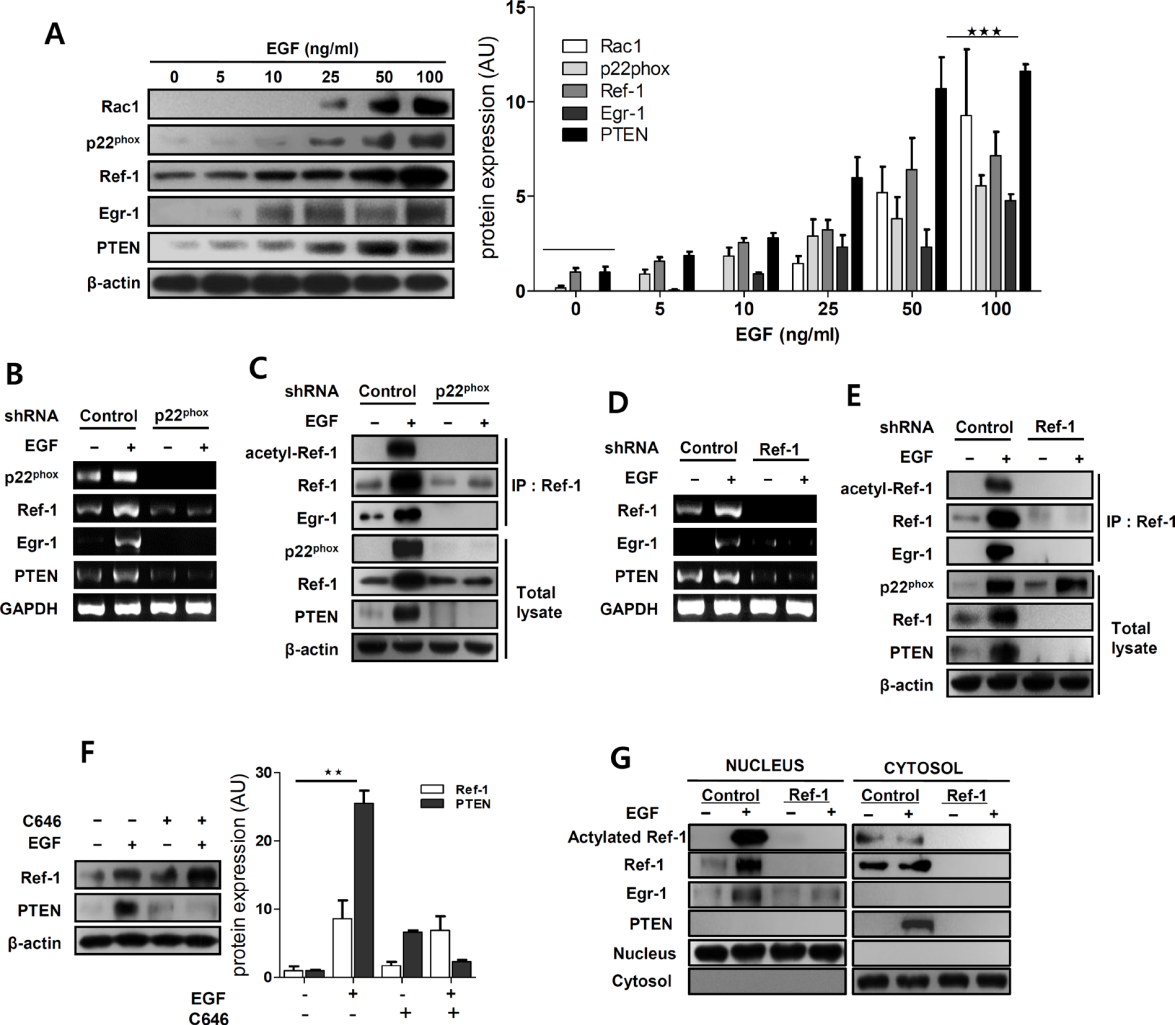
EGF promotes Ref-1 acetylation by regulating redox activity in A549 cells (**A**) The expression of Ref-1-related genes was analyzed using immunoblotting in EGF-treated A549 cells. Data were normalized to β-actin expression. (**B** and **D**) p22^phox^, Ref-1, EGR1, and PTEN mRNA levels were analyzed by RT-PCR in EGF-induced p22^phox^ KD and Ref-1 KD cells. GAPDH was used as an internal control. (**C** and **E**) Immunoprecipitation with anti-Ref-1 antibody was performed using cell lysates from EGF-treated p22^phox^ KD and Ref-1 KD cells. (**F**) After pre-treatment with 1 μM C646, the effects of EGF treatment on PTEN expression were analyzed by immunoblotting. (A and F) Fold-changes are presented in the bar graph. Data are representative of three independent experiments and were analyzed using unpaired *t*-tests (**P < 0.01, ***P < 0.001). (**G**) Representative results of western blot analysis for Ref-1, acetylated Ref-1, EGR1, and PTEN in nuclear and cytoplasmic extracts from EGF-treated control KD and Ref-1 KD cells.

Ref-1, which is induced by oxidative stress that activates transcription factors related to redox signaling [22, 23, 27] can promote either cell survival or death [36, 37]. Ref-1 target genes were measured using western blotting to examine how upregulation of Ref-1 by EGF might inhibit cell growth in A549 cells. EGF treatment markedly increased p22^phox^, Ref-1, EGR1, and PTEN protein levels in a dose-dependent manner (Figure 2A). We then generated p22^phox^ KD and Ref-1KD cells to further investigate how the p22^phox^ NADPH oxidase subunit and Ref-1 affect expression of EGR1 and the tumor suppressor PTEN. Knockdown of p22^phox^ completely reversed EGF-induced increases in Ref-1, EGR1, and PTEN expression (Figure 2B–2C). In addition, EGR1 and PTEN expression did not change in Ref-1 KD cells after EGF treatment, despite normal p22^phox^ expression (Figure 2D–2E). Acetylated Ref-1 activates EGR1 and PTEN [26–28] and levels of acetylated Ref-1 and acetylated Ref-1/EGR1 complexes were higher in EGF-treated A549 cells (Figure 2C and 2E). However, PTEN expression was abolished and acetylated Ref-1/Egr-1 complex levels were negligible in p22^phox^ KD cells (Figure 2C). Ref-1 expression and acetylation were also negligible in Ref-1 KD cells (Figure 2E). We then pre-treated A549 cells with C646, a specific inhibitor of P300 [38], to determine whether the p300/CBP histone acetyltransferase [39] might catalyze Ref-1 acetylation and thereby directly influence EGR1 and PTEN expression. Ref-1 expression was not involved in C646-dependent restoration of EGF-induced PTEN expression (Figure 2F). Finally, flow cytometry using DCH_2_FDA was performed to determine whether ROS-induced increases in Ref-1 increase PTEN expression. Intracellular ROS levels increased 24–72 h after EGF treatment in A549 cells (Supplementary Figure S4) and were reversed to normal levels in p22^phox^KD cells (Supplementary Figure S5). We also examined EGF-induced changes in the cellular localization of Ref-1 protein, which translocate to the nucleus in response to increases in ROS [18, 40, 41], using western blot analysis in control KD and Ref-1 KD cells. Ref-1 and acetylated Ref-1 levels increased in the nuclear compartment in EGF-treated control KD cells (Figure 2G). In addition, EGR1 expression increased in the nuclear compartment in EGF-treated control KD cells (Figure 2G). These findings suggest that acetylation of Ref-1, which increased in response to EGF-induced, p22^phox^-dependent Ref-1 expression, may be associated with EGR1 activation and increased PTEN expression.

### EGF activates Ref-1 by activating P2Y in A549 cells

**Figure 3:**
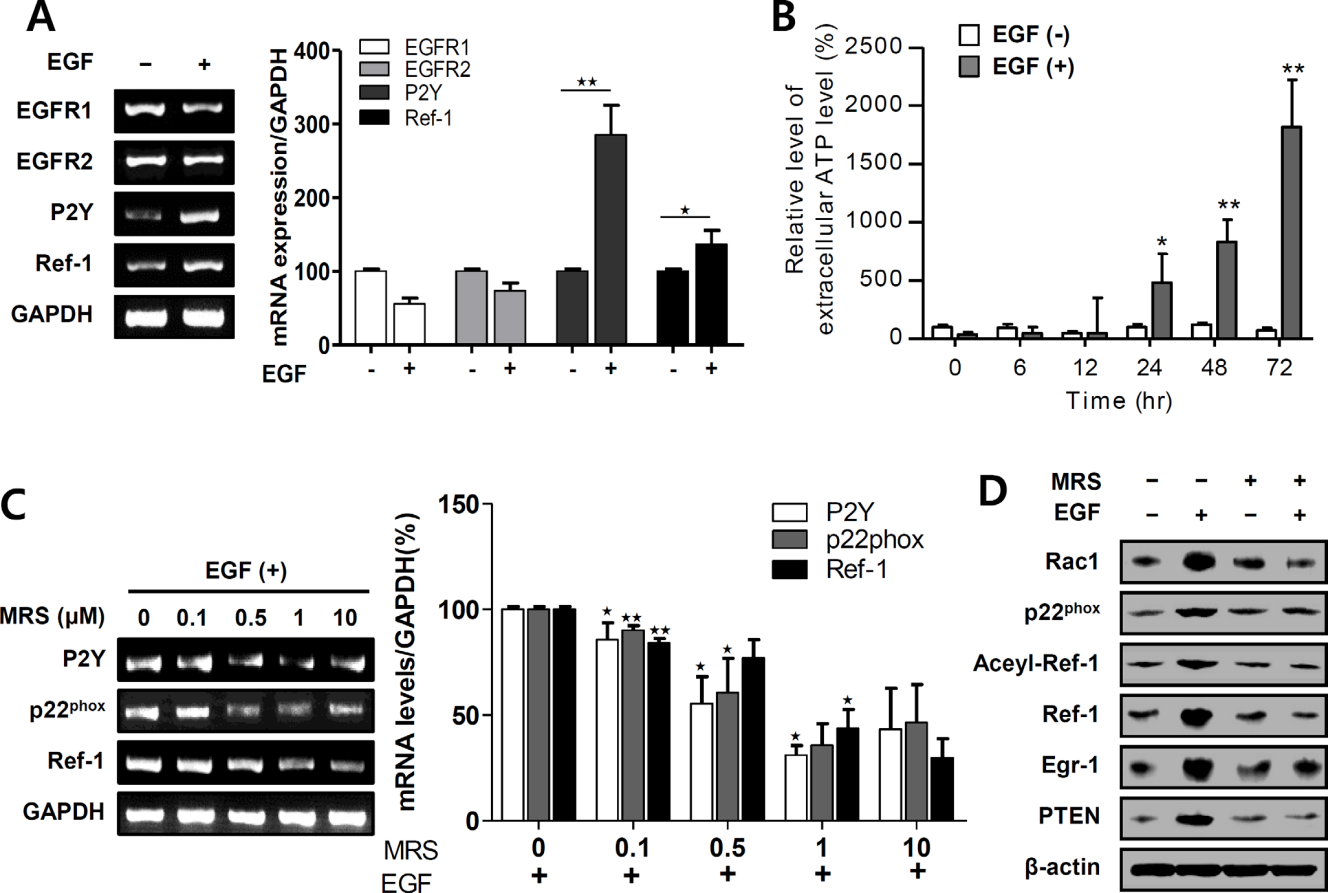
EGF activates P2Y in EGFR-expressing A549 cells (**A**) EGFR1, EGFR2, P2Y, and Ref-1 expression levels were analyzed by RT-PCR in EGF-treated A549 cells. (**B**) Extracellular ATP release was measured using a luminometer (*n* = 8). (**C** and **D**) EGF-induced changes in mRNA and protein levels were measured by RT-PCR and immunoblotting after pretreatment with 1 μM MRS 2500. (A–C) Quantification was carried out using Quantity One software, and results are present as means ± SDs. Data were normalized to control gene levels. Data are representative of three independent experiments and were analyzed using unpaired *t*-tests (*P < 0.05, **P < 0.01).

ATP-P2Y activates NADPH oxidase via RAC1 and, consequently, increases translocation of Ref-1 from the cytosol to the nucleus [18]. We used RT-PCR to measure changes in P2Y expression after EGF-induced Ref-1 upregulation; EGF treatment increased P2Y mRNA levels (Figure 3A). To examine the mechanism underlying this change, we measured secreted extracellular ATP levels in EGF-treated A549 cells and found it to be significantly altered after 24 in EGF-treated A549 cells (Figure 3B). However, intracellular ATP levels did not change in EGF-treated A549 cells (Supplementary Figure S6). To determine whether P2Y activation was directly related to specific EGF-induced genes that lead to cell growth arrest, we measured levels of mRNA transcribed from these genes in cells that were pre-exposed to the P2Y antagonist MRS 2500. MRS 2500 decreased EGF-induced p22^phox^, Ref-1, and P2Y mRNA levels in a dose-dependent manner in A549 cells (Figure 3C). Moreover, MRS 2500 treatment abolished EGF-induced increases in RAC1, p22^phox^, Ref-1, acetylated Ref-1, EGR1, and PTEN expression (Figure 3D). Overall, these data indicate that EGF stimulated intracellular P2Y signaling by releasing extracellular ATP and P2Y, which may in turn contribute to the activation of p22^phox^ and Ref-1.

### EGF inhibits Akt phosphorylation, increases PTEN expression, and inhibits growth in A549 cells

**Figure 4:**
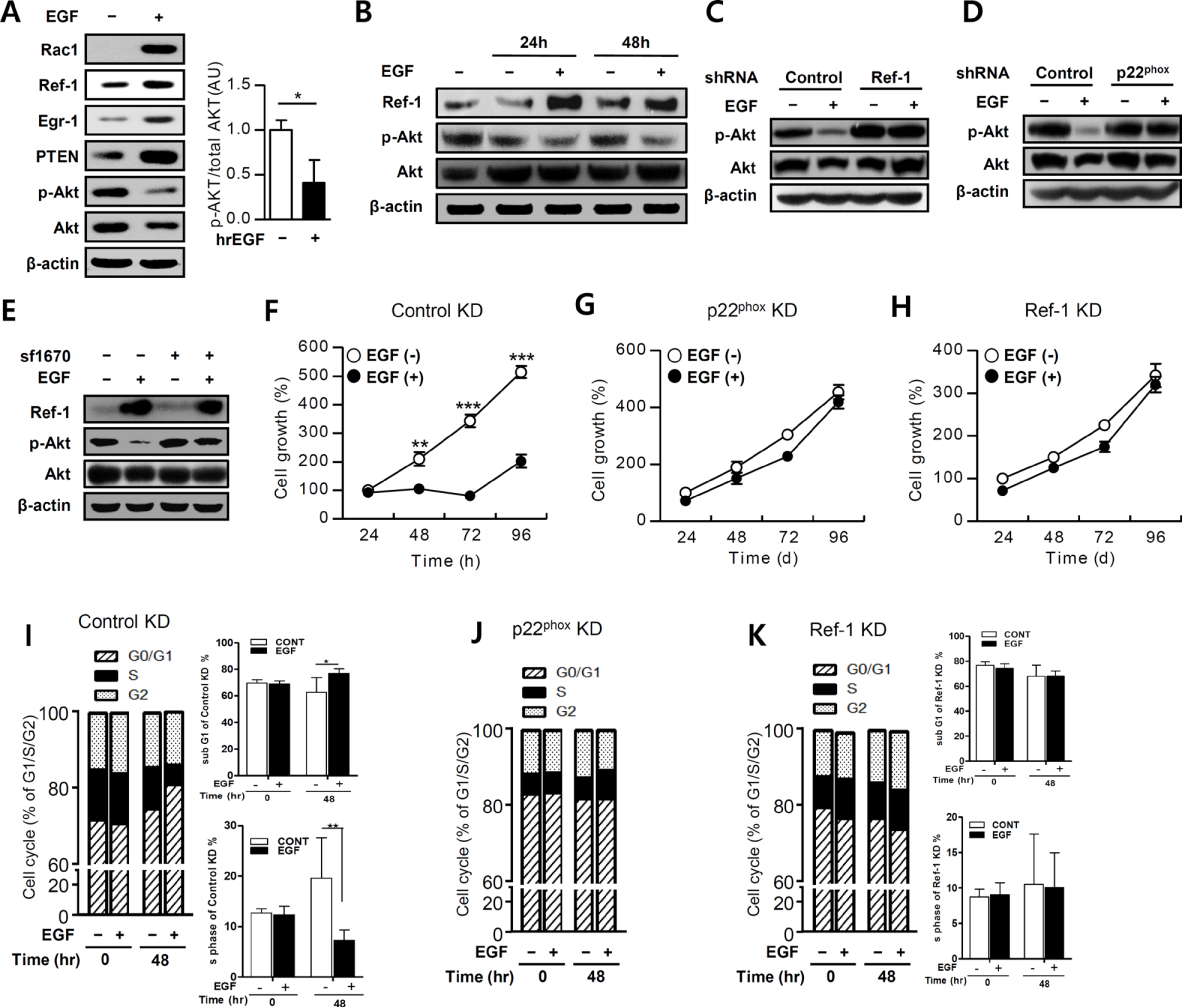
EGF suppresses the Akt pathway in growth-arrested A549 cells (**A** and **B**) EGF-treated A549 cells were incubated with antibodies and then analyzed by immunoblotting. Phospho-Akt levels were normalized to total Akt expression (*n* = 3). (**C** and **D**) Akt activation was measured by immunoblotting in EGF-treated p22^phox^ KD and Ref-1 KD cells. (**E**) Akt activation in cells pretreated with SF1670 (10 μM) was measured by immunoblotting. (**F**–**H**) Growth rates were measured in control KD, p22^phox^ KD, and Ref-1 KD cells using MTT assays after treatment with 100 ng/mL EGF (*n* = 8). Cell cycle progression was analyzed in EGF-treated control KD (**I**), p22^phox^ KD (**J**), and Ref-1 KD cells (**K**) using propidium iodide staining (*n* = 8). Data represent three independent experiments and were analyzed using unpaired *t*-tests (*P < 0.05, **p < 0.01, ***p < 0.001).

EGR1-induced PTEN expression directly inhibits the PI3K/Akt/mTOR pathway in tumor cells [3, 42]. We therefore measured Akt phosphorylation using western blotting during growth inhibition in EGF-treated A549 cells. Treatment with 100 ng/mL EGF increased RAC1, Ref-1, EGR1, and PTEN expression and markedly inhibited Akt phosphorylation in these cells (Figure 4A). EGF treatment blocked Akt phosphorylation over a 24 to 48 h period, while Ref-1 expression markedly increased, in A549 cells (Figure 4B). Furthermore, knockdown of Ref-1 or p22^phox^ reversed EGF-induced inhibition of Akt phosphorylation (Figure 4C and 4D).

To determine whether EGF-induced PTEN expression actively suppresses Akt phosphorylation, we pretreated A549 cells with SF 1670, a specific PTEN inhibitor, which restored EGF-induced Akt phosphorylation (Figure 4E). This suggested that PTEN directly inhibited Akt activation. MTT and cell cycle assays showed that control KD cells and normal A549 cells had similar growth patterns (Figure 1A). Growth differences in the EGF-treated control KD cells were significantly attenuated after 48 h when compared to untreated cells (Figure 4F). Over 24 to 96 h, S phase was decreased and G0/G1 phase was significantly increased in EGF treated-control KD cells, compared to untreated cells (Figure 4I and Supplementary Figure S7). In contrast, EGF treatment did not affect cell growth or cell cycle progression in p22^phox^ KD or Ref-1 KD cells compared to untreated cells (Figure 4G, 4H, 4J, and 4K). Taken together, these results demonstrate that EGF-induced inhibition of cell growth is a result of the Ref-1-induced, p22^phox^-dependent increase in PTEN expression, which subsequently suppresses Akt phosphorylation.

### EGF induces autophagic death in A549 cells

**Figure 5:**
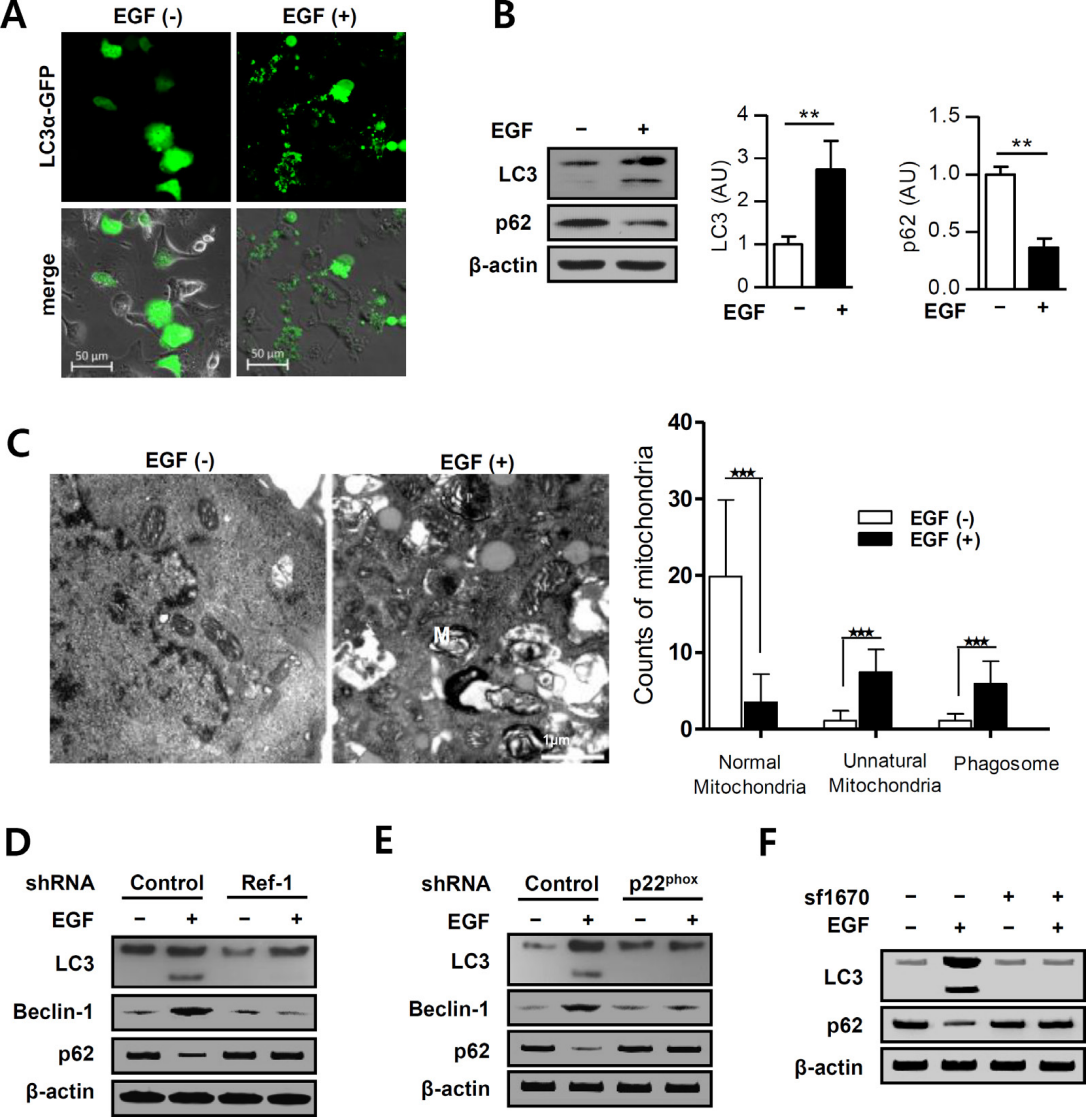
EGF promotes autophagy in A549 cells (**A**) pcDNA3.1-LC3α-GFP-overexpressing A549 cells were incubated with EGF (100 ng/mL). Small puncta of green fluorescence indicate the formation of autophagosomes in the cytosol. (**B**) Protein levels were analyzed by immunoblotting (*n* = 3 independent experiments). (**C**) Dysfunctional mitochondria were identified in EGF-treated cells using EM (original magnification, 12,000×; M: mitochondria, P: phagosome). (B and C) Data represent three independent experiments and were analyzed using unpaired *t*-tests (**p < 0.01, ***p < 0.001). EGF treatment induced autophagosome formation in (**D**) Ref-1 KD and (**E**) p22^phox^ KD cells. (**F**) LC3 and p62 expression levels were analyzed by immunoblotting in cells pretreated with SF1670 (10 μM).

PTEN induces autophagy [43]. To examine whether EGF increases the formation of autophagosomes, alterations in LC3 expression were evaluated using a pcDNA-LC3-EGFP vector. EGF treatment increased the formation of GFP-LC3 puncta, which are indicative of autophagosome formation, in A549 cells (Figure 5A). Furthermore, EGF markedly increased the conversion of LC3-I to LC3-II (Figure 5B, 5D–5F). P62/SQSTM1 is a crucial regulator of cell survival and death, and reduced p62 expression can cause autophagy defects and induce autophagic cancer cell death [44, 45]. EGF treatment decreased p62 expression in A549 cells (Figure 5B), and Ref-1 knockdown restored LC3-II and p62 expression in these cells (Figure 5D). p22^phox^ knockdown also inhibited EGF-induced conversion of LC3-I to LC3-II and decreases in p62 expression (Figure 5E). We then pre-treated cells with the PTEN inhibitor SF1670 to determine whether EGF-induced PTEN expression promotes the formation of autophagosomes. SF1670 pre-treatment completely blocked the EGF-induced increase in LC3-II and decrease p62 expression in A549 cells (Figure 5F). These results suggest that Ref-1-induced upregulation of PTEN promoted autophagy. To determine whether the EGF-induced suppression of cell growth was dependent on autophagy, electron microscopy was used to examine A549 cell ultra-structures. Numbers of dysfunctional mitochondria, vacuoles, and mature autophagosomes clearly increased in these cells 24 h after EGF treatment (Figure 5C). These results suggest that continuous EGF exposure triggers PTEN-induced autophagic death in A549 cells by stimulating Ref-1 activity.

### EGF induces apoptotic death in A549 cells

**Figure 6:**
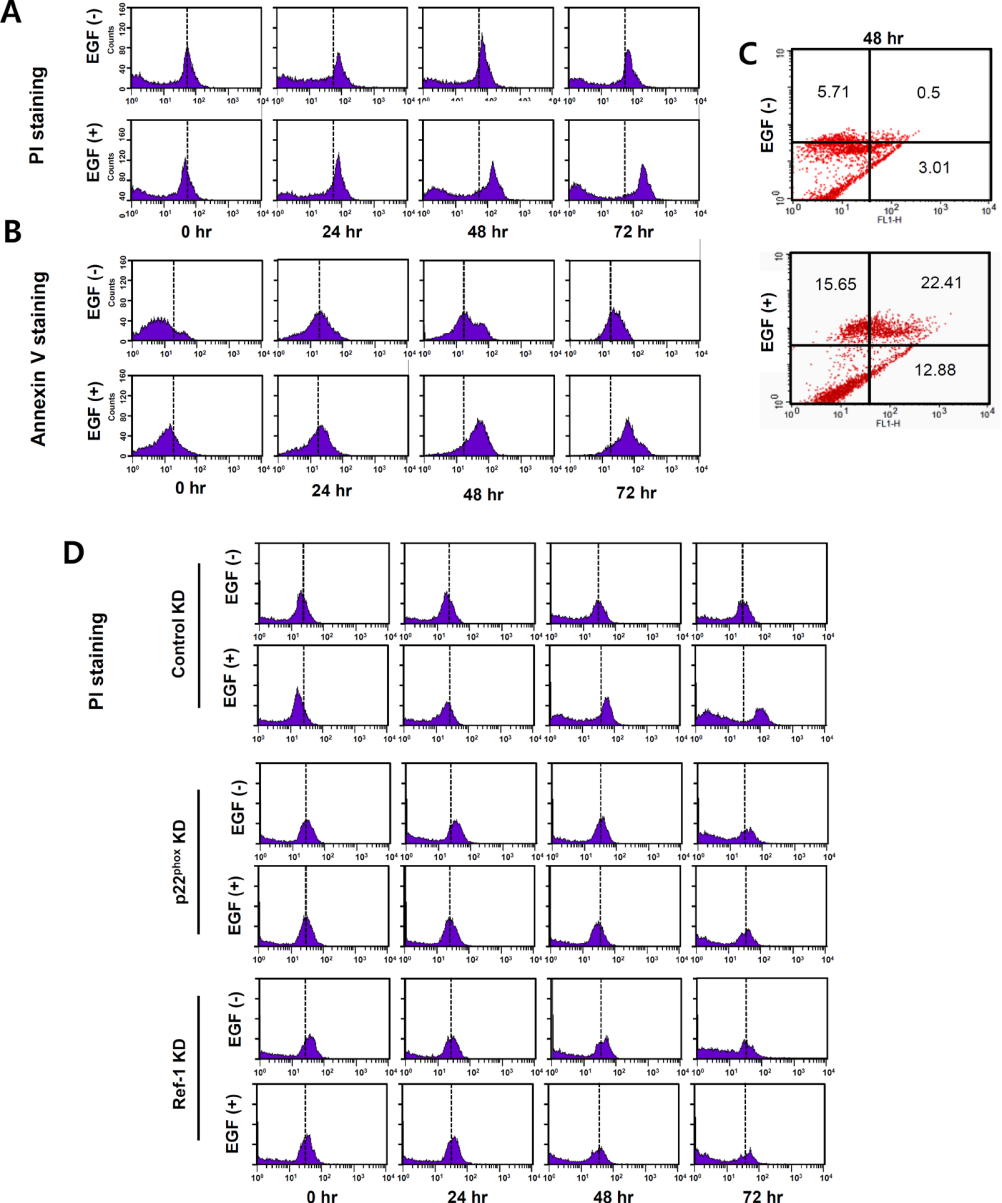
EGF increases apoptosis in A549 cells (**A**–**D**) Apoptosis and cell death were analyzed using flow cytometry with PI and annexin V staining in EGF-treated A549 cells (A–C) and in EGF-control KD, p22^phox^KD, and Ref-1KD cells (D). Representative results of three independent experiments are shown.

EGR1, a pro-apoptotic protein, inhibits growth and increases apoptosis in human cancer cells by upregulating PTEN [26, 27]. To determine whether EGF-induced PTEN expression might lead to apoptosis in A549 cells, Annexin V and PI staining were used to measure cell death. After at least 24 h of exposure to EGF, apoptosis increased after 48 and 72 h (Figure 6A and 6B). Figure 6C shows annexin V and PI co-staining 48 h after EGF treatment. EGF treatment also increased apoptosis in control KD cells (mean PI staining values at 72 h: 15.29 vs. 41.0 and 15.52 vs. 31.29 for control and EGF-treated A549 cells, respectively; Figure 6D). In contrast, cell death as indicated by PI staining was minimal in p22^phox^ KD and Ref-1 KD cells under the same conditions compared to control KD cells (p22^phox^ KD cells mean PI values, 16.28 vs. 16.88, Ref-1 KD cells mean PI values, 13.35 vs. 13.73 for control and EGF-treated cells, respectively, at 72 h; Figure 6D). Collectively, these results revealed that EGF-induced, p22^phox^- and Ref-1-dependent apoptosis and cell death requires more than 24 h.

### EGF induces mitochondrial dysfunction and cell death in A549 xenograft tumors

**Figure 7:**
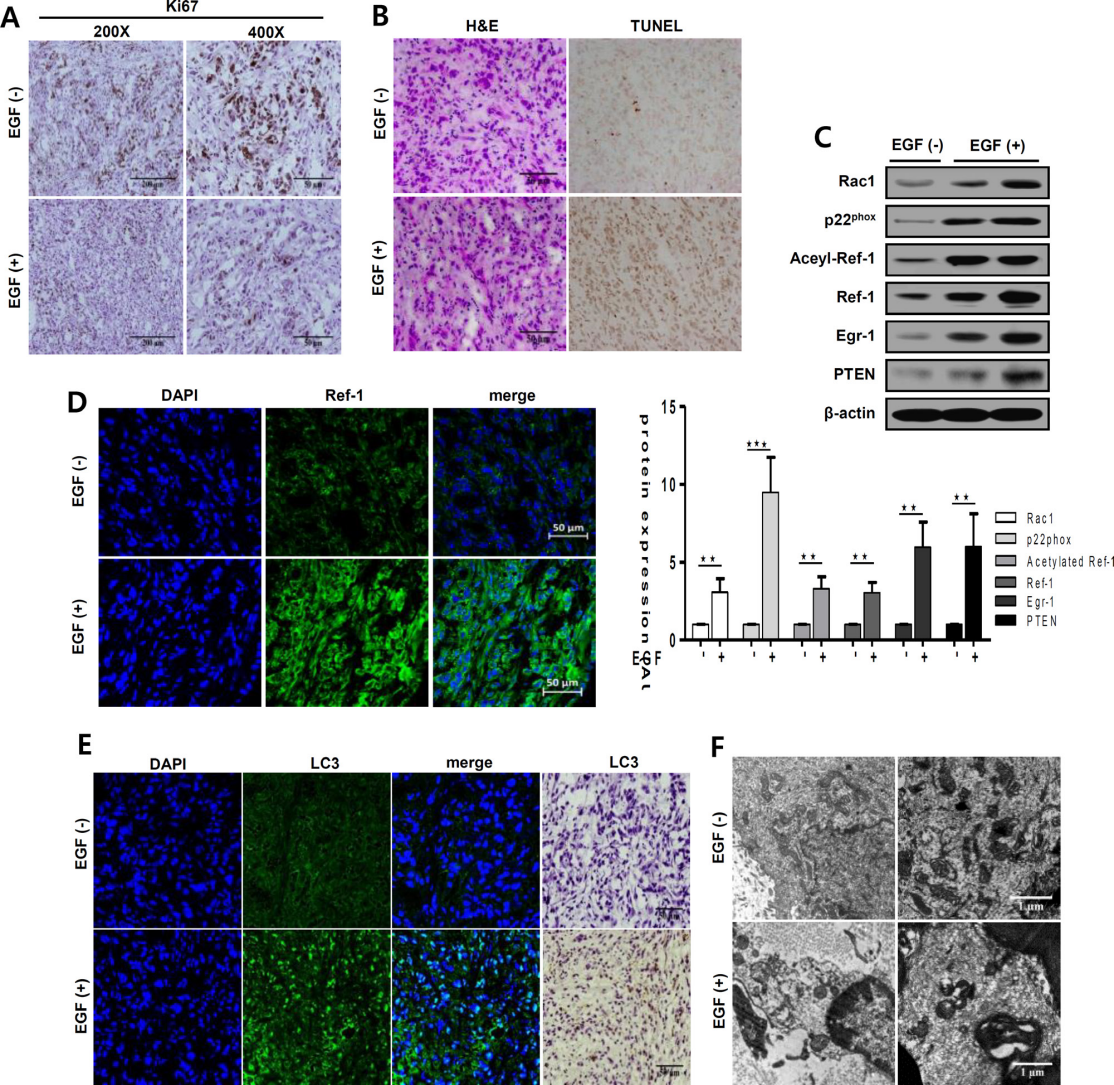
EGF increases apoptosis and autophagy in xenograft tumors Tumor tissues (50 mm^3^) in six-week-old nude mice (*nu*/*nu*) were treated daily with EGF (1 mg/kg) for 21 days. Tumors were separated from normal tissues and isolated from mice injected with PBS (control group) or with 1 mg/kg EGF. (**A**, **B**, and **D**) Tumors were immunohistochemically stained with antibodies against Ki67, a marker of cell proliferation (A) or LC3, a marker of autophagy (**E**). Hematoxylin and eosin was used as a counterstain. Scale bar, 50 μm (A, B, D, and E). (B) EGF-induced changes in tumor cell death were analyzed by TUNEL staining. Dark brown dots represent nuclei of cells undergoing apoptosis. (**C**) RAC1, p22^phox^, acetylated Ref-1, Ref-1, EGR1, PTEN, and β-actin protein levels were analyzed by western blotting. Data are representative of three independent experiments. Quantification was carried out using Quantity One software and showing unpaired *t*-tests (**p < 0.01, ***p < 0.001). (D, E) Tumors were stained with anti-Ref-1 and anti-LC3 antibodies and then with Alexa Fluor 488-conjugated secondary antibodies (green). Nuclei were counterstained with 4′,6-diamidino-2-phenylindole (DAPI, blue). (**F**) Subcellular structures in tumors were examined in detail using electron microscopy (magnification, 7,000×). Scale bar, 1 μm. Quantification was shown in Supplementary Figure S8.

To confirm our findings *in vivo*, we evaluated the effect of EGF on tumor growth in an A549 cell xenograft model of solid lung carcinoma. Body weight and tumor size measurement were shown in Supplementary Figure S9. In both, groups were consistently increased, and EGF had no effect on body weight. Whereas, EGF-injected tumor size was significantly decreased compare with control tumor size. EGF treatment partially inhibited proliferation and induced cell death in tumor tissues (Figure 7A and 7B). To determine whether EGF treatment similarly inhibited tumors *in vivo*, we examined Ki67 expression to assess tumor cell proliferation. Proliferation was suppressed in the EGF-exposed area compared to control tumor tissues (Figure 7A). Simultaneously, cell death increased in EGF-treated tumors (Figure 7B). Moreover, as was the case *in vitro*, RAC1, p22^phox^, EGR1, and PTEN expression were higher in tumor tissues from EGF-treated mice compared to tumor tissues from PBS-treated control mice (Figure 7C). Staining with the anti-Ref-1 FITC-conjugated antibody showed that Ref-1 expression increased dramatically in EGF-injected tumors (Figure 7D). Finally, ICH and IF were performed to assess whether autophagy differed in tumors from EGF-injected groups. As shown in Figure 7E, LC3 expression and LC3 puncta markedly increased in EGF-injected mice compared to the control group. In addition, EM revealed that EGF treatment altered mitochondrial morphology in A549 cells, indicating that autophagic dysregulation increased (Figure 7F and Supplementary Figure S8). These findings suggest that continuous exposure to high levels of EGF inhibited cell growth in this tumor model, which is consistent with our *in vitro* findings.

## DISCUSSION

Here, we found that EGF attenuated growth in NSCLC cells. In particular, EGF inhibited colony formation in NSCLC cells, suggesting that EGF–EGFR signaling exerted novel effects on these cells that have not been previously described. We previously reported that EGF inhibits tumor cell growth, likely by triggering apoptosis [6, 11]. EGF treatment also markedly reduced cell proliferation rates, as indicated by Ki67 immunohistochemistry, in xenograft tumor tissue, indicating that EGF attenuated tumor growth *in vivo* as well as *in vitro*. The ligand EGF antagonizes constitutively expressed mutant EGFR, allowing it to act as a ‘cell death switch’ by altering apoptotic receptor signaling [9, 10, 46]. EGF also induces programmed cell death in tumors that express high levels of EGFR1 and EGFR2 [6–9]. Our findings in EGFR1 KD cells also suggest that EGF-induced inhibition of cell growth in NSCLC cells is dependent upon EGFR expression.

**Figure 8:**
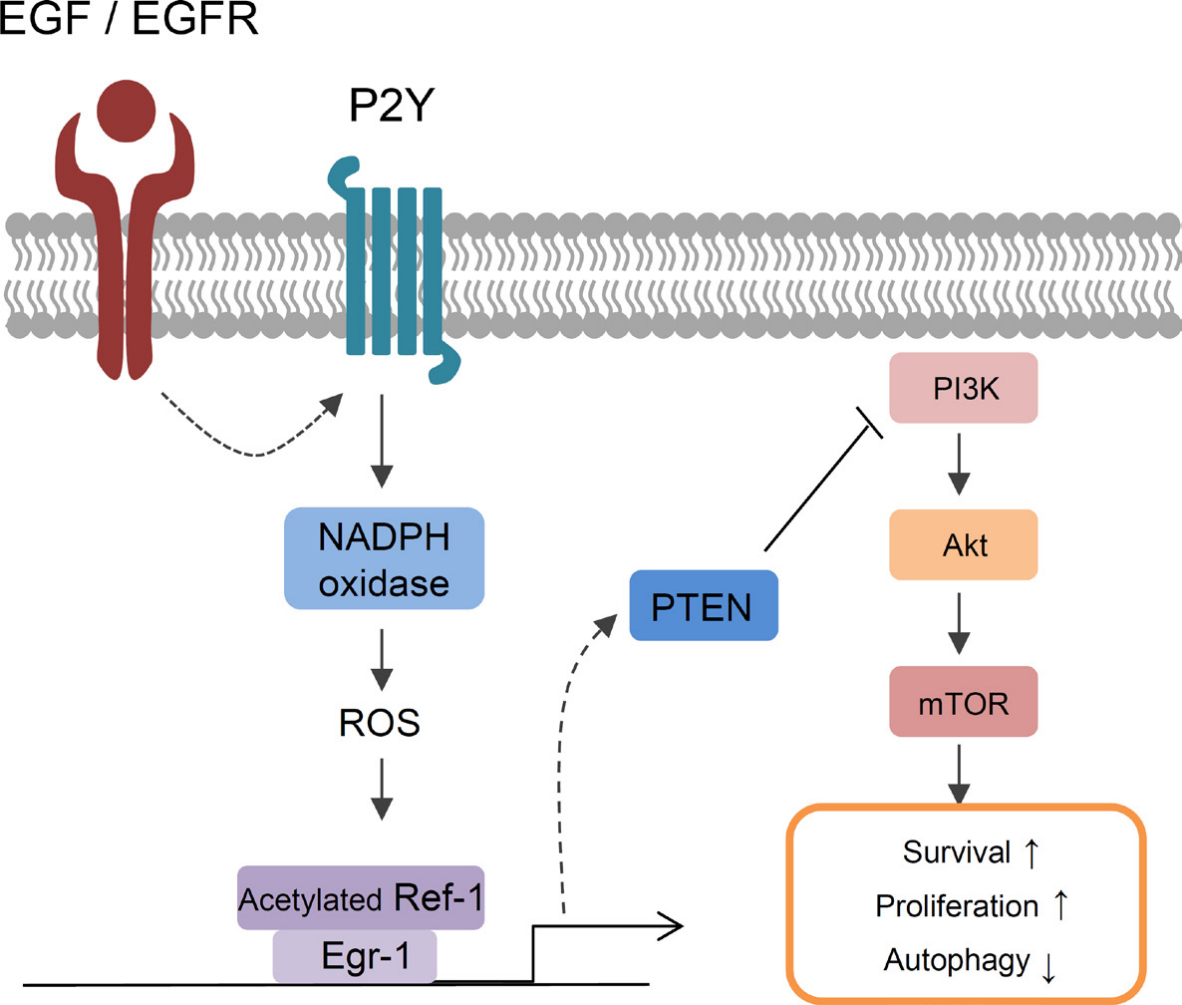
A model of EGF-induced signaling in lung cancer cells EGF/EGFR-induced cell death in NSCLC was associated with acetylation of Ref-1, followed by the formation of Ref-1/EGR1 complexes and increases in PTEN expression. Constitutive activation of EGFR in response to EGF treatment increased P2Y-dependent NADPH oxidase-induced oxidative stress. ROS induced the acetylation of Ref-1, which then translocated to the nucleus. Increased EGR1 expression during oxidative stress also increased the formation of acetylated Ref-1/EGR1 protein complexes. Activated Ref-1/EGR1 complexes increased PTEN expression and inhibited the phosphorylation of Akt. Together, these processes promoted apoptosis and autophagic dysfunction in NSCLC cells.

Here, we found that EGF-EGFR signaling mediates NSCLC cell survival based on the following novel findings: (i) continuous treatment with EGF markedly increased the expression of RAC1 and p22^phox^, which are major subunits of NADPH oxidase, and Ref-1, which regulates redox reactions during oxidative stress, in an EGFR1-dependent manner; (ii) EGR1 increased levels of acetylated Ref-1 and the Ref-1/EGR1 complex, which induced PTEN-mediated programmed cell death; (iii) EGF increased extracellular ATP release in A549 cells; and (iv) EGF increased the expression of P2Y, a G-coupled protein receptor (GPCR) that belongs to the purinergic receptor family and responds to changes in extracellular ATP levels. We found that the levels of specific *P2Y*1 mRNA were increased by EGF treatment. Additionally, prior EGF, pretreatment with specific P2Y_1_ antagonist (MRS 2500) abrogated EGF-induced genes expression. We evaluated whether signaling through the P2Y_1_ subtype was involved in EGF-mediated ROS production, Ref-1/EGR1 activation, and PTEN upregulation in A549 cells. RAC1 is well-known for increasing ROS production via the EGF–EGFR pathway [12, 13, 26]. RAC1 and p22^phox^ form an essential subunit of all functional, activated NADPH oxidase isotypes [47]. In the absence of the p22^phox^ protein, NADPH oxidase loses its enzymatic activity, which prevents ROS production [48, 49]. p22^phox^ knockdown abolished both NADPH oxidase activity and EGF-induced PTEN expression, suggesting that EGF-EGFR-induced oxidative stress resulting from intracellular ROS production is required for EGF-mediated increases in gene expression, including Ref-1, EGR1, and PTEN. Taken together, these results suggest that activated Ref-1 may induce EGR1- and PTEN-mediated programmed cell death by activating EGF–EGFR-induced, P2Y-NADPH oxidase-mediated ROS production (Figure 8).

EGF–EGFR signaling can promote either cell survival or death [9, 10]. Our present findings revealed that Ref-1 and activated Ref-1 are key inducers of EGF-EGFR-mediated cell death. Indeed, subcellular localization of Ref-1 has been associated with higher mortality and poor prognosis in NSCLC patients [24, 25, 50]. When activated EGFR binds to EGF, it increases PTEN expression by activating Ref-1. Ref-1 stimulates the cellular response to acute and chronic oxidative stress and can control intracellular ROS levels by inhibiting ROS production. Ref-1 has separate C-terminal functional domains that contribute to DNA repair and its N-terminal controls redox activity by directly regulating various transcriptional factors. Due to its unique structure, Ref-1 can affect many cellular processes, including cell cycle arrest, apoptosis, and cell survival [22, 23]. Ref-1 does not act as a transcription factor on its own, but affects cancer cell survival by directly affecting the expression of important redox-transcription factors in response to changes in the intracellular environment in a cell type-specific manner [22, 25, 27, 51].

In response to oxidative stress, Ref-1-associated EGR1 activates the tumor suppressor PTEN, leading to cell cycle arrest and programmed cell death [26–28]. Actively progressive malignant cancers exhibit loss of PTEN expression, which results in the dysregulation of multiple Akt-dependent and -independent pathways [52]. Moreover, loss of PTEN expression causes strong resistance to EGFR-targeting drugs, such as gefitinib or erlotinib, by preventing the suppression of Akt activation and promoting EGR1 translocation in NSCLC cells [2, 53, 54]. Ref-1 knockdown abolished EGF-induced increases in EGR1 and PTEN protein levels. In addition, EGF markedly induced Ref-1 expression and posttranslational Ref-1 acetylation; these effects might be a direct result of increased PTEN expression. Ref-1-induced EGR1 expression in turn upregulates PTEN expression, which promotes apoptosis [27, 55]. Here, we found that EGF–EGFR signaling increased Ref-1, EGR1, and PTEN expression. p22^phox^ and Ref-1 knockdown reversed these decreases in cell survival, regardless of whether or not cells were treated with EGF. In this study, p22^phox^ and Ref-1 activation increased EGF-induced inhibition of Akt phosphorylation. Furthermore, pretreatment with the PTEN inhibitor SF1670 restored Akt phosphorylation, suggesting that EGF-induced increases in PTEN expression might have a profound effect on cell fate.

We also found that EGF induced autophagic cell death both *in vivo* and *in vitro*, as indicated by increases in mitochondrial dysfunction that characterize this process. PTEN or oxidative stress can increase autophagy in both PI3K/Akt/mTOR pathway inhibition-dependent and -independent manners [56, 57]. In this study, EGF-induced increases in LC3 activation was dependent on p22^phox^ and Ref-1 activation, and we previously observed that increasing PTEN expression and inhibiting Akt phosphorylation may be important for the initial stages of autophagy. Taken together, these findings indicate that ROS-mediated Ref-1 activation via EGF–EGFR signaling might play a critical role in various pathways, including activating EGR1 and PTEN expression, cell growth arrest, autophagy, and apoptosis. We obtained similar results *in vitro*, in which newly activated LC3 coincided with dysfunctional mitochondria and DNA damage in EGF-injected tumors, suggesting that the EGF–EGFR network also induced cell death via Ref-1 activation in a xenograft tumor model.

Because EGF–EGFR signaling can promote either cell survival or death, it is an oversimplification to state that EGFR activity increases survival in cancer cells. Here, we report that EGF–EGFR signaling was closely connected with Ref-1 activity. Although Ref-1 is a single gene, it can lead to either cell death or survival under conditions of intracellular or environmental stress. A better understanding of how Ref-1 effects the balance between cellular death and survival signals could lead to its use as a predictive marker for therapeutic response and resistance to EGFR-targeting treatments in lung cancer patients.

## MATERIALS AND METHODS

### Cell culture, animals, and reagents

NSCLC cells (A549, EKVX, NCI-H23, NCI-H226, NCI-H322M, and NCI-H522) were obtained from the NCI-Fredrick Cancer Center and were routinely cultured in RPMI-1640 medium (Gibco BRL) for 4–6 passages. Short tandem repeats profiling was used to confirm the identity of the NSCLC cells (Supplementary Table S1, S2). Immunodeficient mice (*nu*/*nu*; Harlan Sprague–Dawley, Inc. Indianapolis, IN) were maintained under pathogen-free conditions at the animal resources center (ARC) at Asan Medical Center, and all animal studies were conducted in accordance with the Institutional Animal Care and Use Committee (IACUC) guidelines. Mice used for xenograft experiments were euthanized by CO2 inhalation (30% vol/min). Human recombinant epidermal growth factor (EGF, 236-EG, R&D systems, USA) protein, C646 (SML 0002-5MG, SIGMA Life Science), SF1670 (SML 0684-5MG, SIGMA Life Science), and MRS 2500(2159, TOCRIS Bioscience) were used for experimental treatments

### Generation of cell lines with stable knockdown using a lentiviral shRNA system

Bacteria glycerol stocks of pLKO.1-puro-human CYBA (p22^phox^), -human APEX1 (Ref-1), and –human EGFR1 and -U6-EGFP control lentiviral plasmid vectors were purchased from Sigma–Aldrich. 293FT cells (Invitrogen) were co-transfected with both the vectors and plasmids encoding lentiviral-coating proteins (pMDLG/pRRE, pRSV-REV, and pMD.G) for 36 h. Viral particles were then concentrated from 293FT host cells using a Lenti-X^™^ Concentrator (Clontech). A549 cells were injected with the particles to produce cell lines with stable knockdown of the following target genes: shU6-control, shU6-EGFR, shU6-p22^phox^, and shU6-Ref-1. After four passages of A549 cells selected using puromycin (BML-GR312, ENZO Life Science), stable and specific knockdown of the target gene was confirmed using reverse-transcriptase-polymerase chain reaction (RT-PCR) and western blotting.

### Cell viability assays

The 3-(4,5-dimethylthiazol-2-yl)-2,5-diphenyl-tetrazolium bromide (MTT) assay (TACS MTT kit, #4890-25-02, TREBIGEN^®^ Instruction) was used to assess cell viability in a proliferation assay context according to the manufacturer's instructions and as described previously [6].

### Colony formation assay

Untreated control and EGF-pretreated cells were plated at a low density (300 ~ 600 cells/well) in RPMI-1640 media. Fresh medium containing EGF (100 ng/mL) was added once every three days. After two weeks of culture, cells were fixed with 4% formaldehyde for 10 min and stained with crystal violet (0.05%, 2 h).

### Measurement of mRNA expression

Total RNA was extracted from A549 cells using TRIzol reagent (Invitrogen), and 1 μg total RNA from each sample was transcribed. RT-PCR, consisting of 27–30 cycles at 94°C for 30 sec, 58°C for 50 sec, and 72°C for 1 min, was performed using the following specific primers: human *P2Y* (NM_002563.4), human *EGFR1* (NM_005228.3), human *EGFR2* (NM_004448.3), human *Ref-1* (S43127.1), human *EGR1* (NM_001964.2), and human *PTEN* (NM_000314.6). As an internal control, the mRNA transcript for human *GAPDH* (BC083511.1) was amplified and analyzed under identical conditions using a pair of specific primers (primer sequences; Supplementary Table S2).

### Adenosine 5′-triphosphate (ATP) measurements

Extracellular ATP was measured according to the manufacturer's protocol (Promega Corp. ENLITEN^®^ ATP assay system bioluminescence detection kit #TB267). Samples were quantitatively measured using a VICTOR3 (multilabel readers; PerkinElmer).

### Western blotting and immunoprecipitation (IP)

Nuclear and cytoplasmic protein was extracted and fractionated as previously described [9]. Western blotting was performed using the following antibodies: Ref-1 (E-17, 1:250, Cat sc-9919, and C-4, 1:250, Cat sc-17774, Sana Cruz), Acetylated Ref-1 (1:1000, Cat D12045, Ancell), p22^phox^ (FL-195, 1:200, Cat sc-20781, Santa Cruz), Beclin-1 (D40C5, 1:1000, Cat #3495, Cell Signaling), LC3B ((D11)XPTM, 1:1000, Cat #3836, Cell Signaling), p62 (D5E2, 1:1000, Cat #8025, Cell Signaling), EGR1 (15F7, 1:250, Cat #4153, Cell Signaling), PTEN (138G6, 1:500, Cat #9559, Cell Signaling), Akt (pan; C67E7, 1:1000, Cat #9916, Cell Signaling), phospho-Akt (ser473, Cat #9916, 1:1000, Cell Signaling), RAC1 (1:200, Santa Cruz), and β-actin (1:5000, Santa Cruz) as described previously [6]. Samples from A549, control KD, p22^phox^ KD, and Ref-1 KD cells were also used for immunoprecipitation, which was performed as described previously [48].

### Cell cycle analysis

A549, control KD, p22^phox^ KD, and Ref-1 KD cells were treated with 100 ng/mL EGF, and cell cycle analysis was performed using a flow cytometer as described previously [39].

### Immunofluorescence (IF) and immunohistochemistry (IHC)

Tumor samples from xenograft mice were stained with the following antibodies: Ki67 (SP-6, 1:250, Cat ab16667, Abcam), LC3B ((D11) XPTM, 1:250, Cat #3836, Cell Signaling), and Ref-1 (C-4, 1:200, Cat sc-17774, Sana Cruz) as described previously [48, 58]. IF images were collected with a single rapid scan using a LSM 710 confocal microscope (Zeiss). IHC images were obtained using an Olympus DP71 microscope and Olympus DP controller software.

### Electron microscopy (EM)

A549 cells were treated with EGF for 48 h, and xenograft tumor samples were fixed with 2% glutaraldehyde in 0.1 M phosphate buffer. Samples embedded in super resin were cut into 60 nm sections using an ultra-microtome. Sections were stained with saturated solutions of uranyl acetate and lead citrate and were visualized using an electron microscope.

### Detection of apoptosis

A549, control KD, p22^phox^ KD, and Ref-1 KD cells were treated with 100 ng/mL EGF for 0, 24, 48, or 72 h. Cells were then stained with Annexin V-FITC antibody and propidium iodide (PI) according to the instructions provided with the Apoptosis Detection Kit I (BD Pharmingen^™^). Apoptosis was analyzed using flow cytometry (FACS Scan and CELLQUEST software; BD Biosciences).

### TUNEL assay

TdT-mediated dUTP-fluorescein nick-end labeling (TUNEL) assays were performed according to the manufacturer's instructions (Promega Corp. DeadEnd^™^ Colorimetric TUNEL System #G7132). Images of random fields in tumor tissues were captured using an Olympus DP71 microscope and percentages of terminal transferase-labeled cells were determined using Olympus DP controller software.

### Statistical analysis

Results are shown as means ± standard deviations (SD) of between three and eight independent experiments. Means for different groups were compared using unpaired, two-tailed Student's *t*-tests (*p < 0.05; **p < 0.01; ***p < 0.001).

## SUPPLEMENTARY FIGURES AND TABLES


