# Supplementary material for: Paradoxical induction of growth arrest and apoptosis by EGF via the up-regulation of PTEN by activating Redox factor-1/Egr-1 in human lung cancer cells
Source: Oncotarget. 2016 Dec 7;8(3):4181–95. doi: 10.18632/oncotarget.13809 (PMC5354822; doi:10.18632/oncotarget.13809)
Supplement: Supplementary file 1 [file oncotarget-08-4181-s001.pdf]

Paradoxical induction of growth arrest and apoptosis by EGF via the up-regulation of PTEN by activating Redox factor-1/Egr-1 in human lung cancer cells

Supplementary Materials

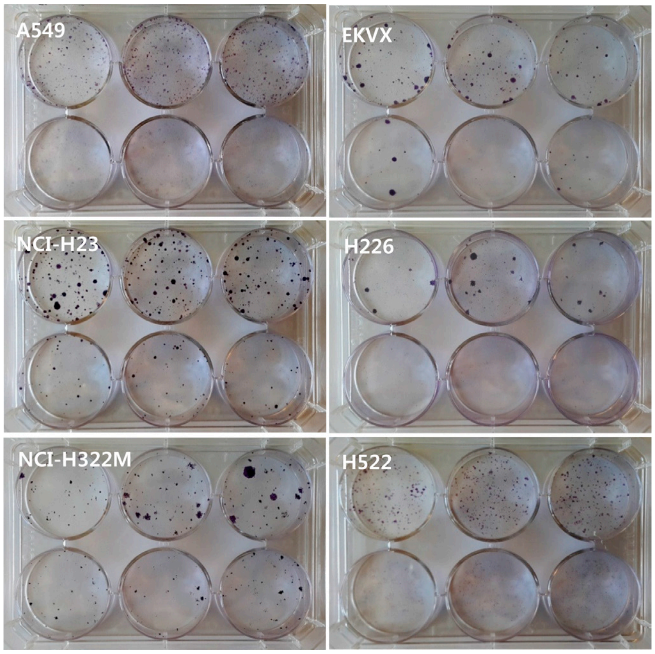

Supplementary Figure S1: Effect s of EGF on the growth rate of NSCLC cells. Colony formation is shown in six lung cancer cell lines (A549, EKVX, NCI-H23, NCI-H266, NCI-H322M, and NCI-522). Triplicate wells were treated with 0 or 100 ng/mL EGF with PBS once every 3 days for 15days.

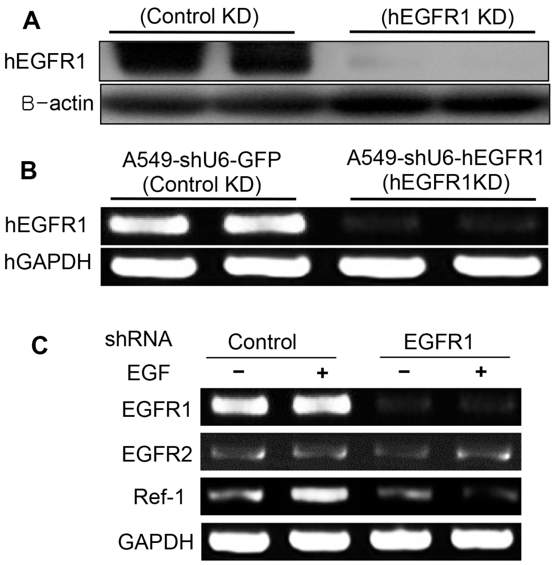

Supplementary Figure S2: EGFR1 and Ref-1 mRNAs were abolished in EGFR1 knock down (KD) cells. A. Stable A549 cells with shRNA-mediated knockdown of EGFR1 using the shRNA-Lentiviral system(EGFR1KD) were evaluated by western blotting. B and C. EGFR1KD cells were detected by RT-PCR using gene-specific primers (EGFR1, EGFR2, and Ref-1). GAPDH was used as an mRNA loading control.

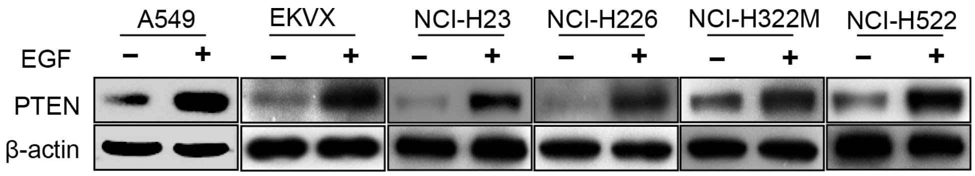

Supplementary Figure S3: PTEN expression levels in NSCLC cell lines. Six NSCLC cell lines were incubated with 100ng/mL EGF for 24 h, and the expression levels of PTEN and β-actin were analyzed by western blotting.

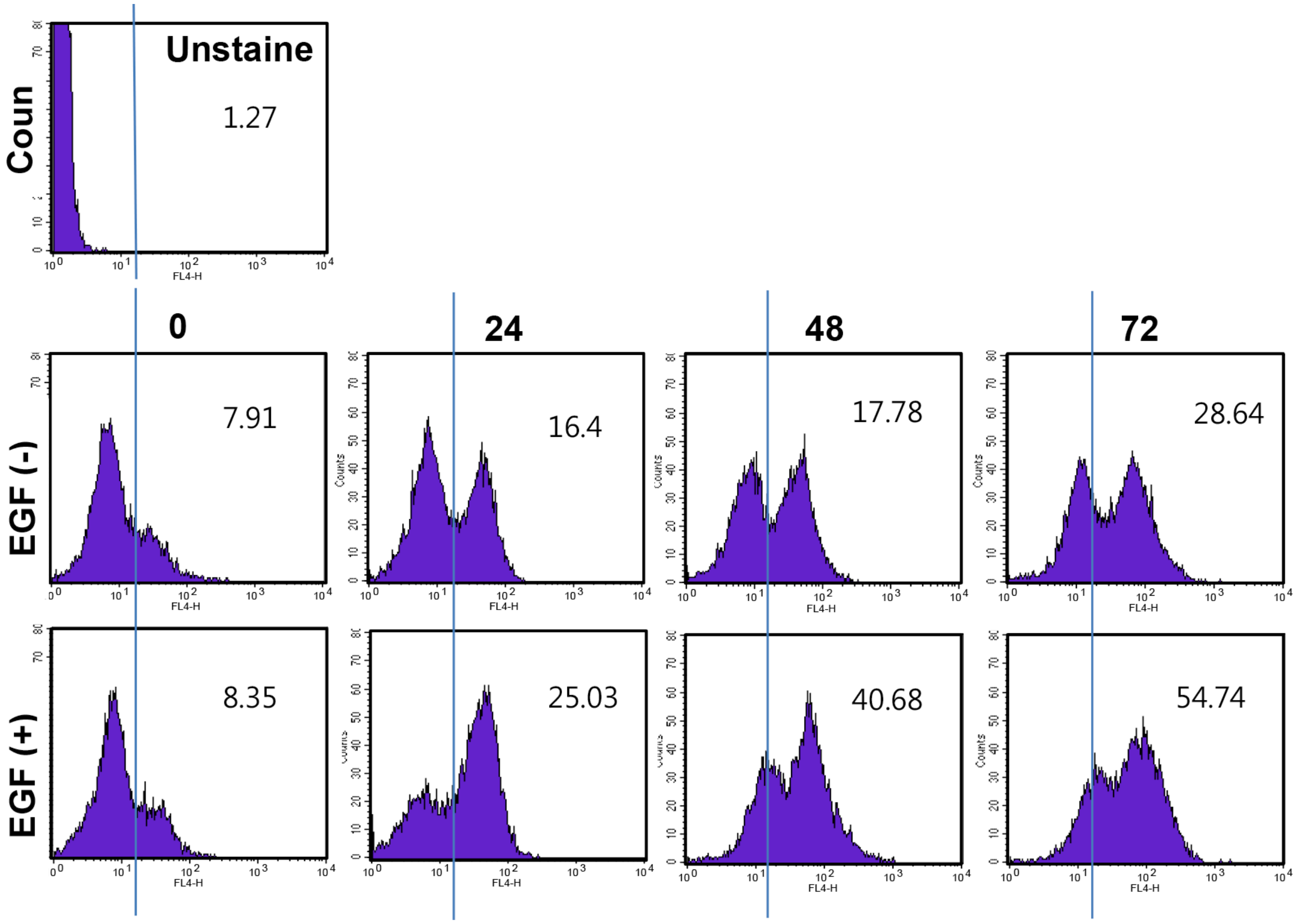

Supplementary Figure S4: Intracellular ROS was increased after EGF treatment in A549 cells. A total of 5 × 105 cells/well was incubated with 100 ng/mL hrEGF for 0, 24, 48, or 72 h. The percentage of cells in the upper section of the histogram indicates normal cells with little or no fluorescence, whereas the lower side indicates cells with higher fluorescence, representing increased ROS generation.

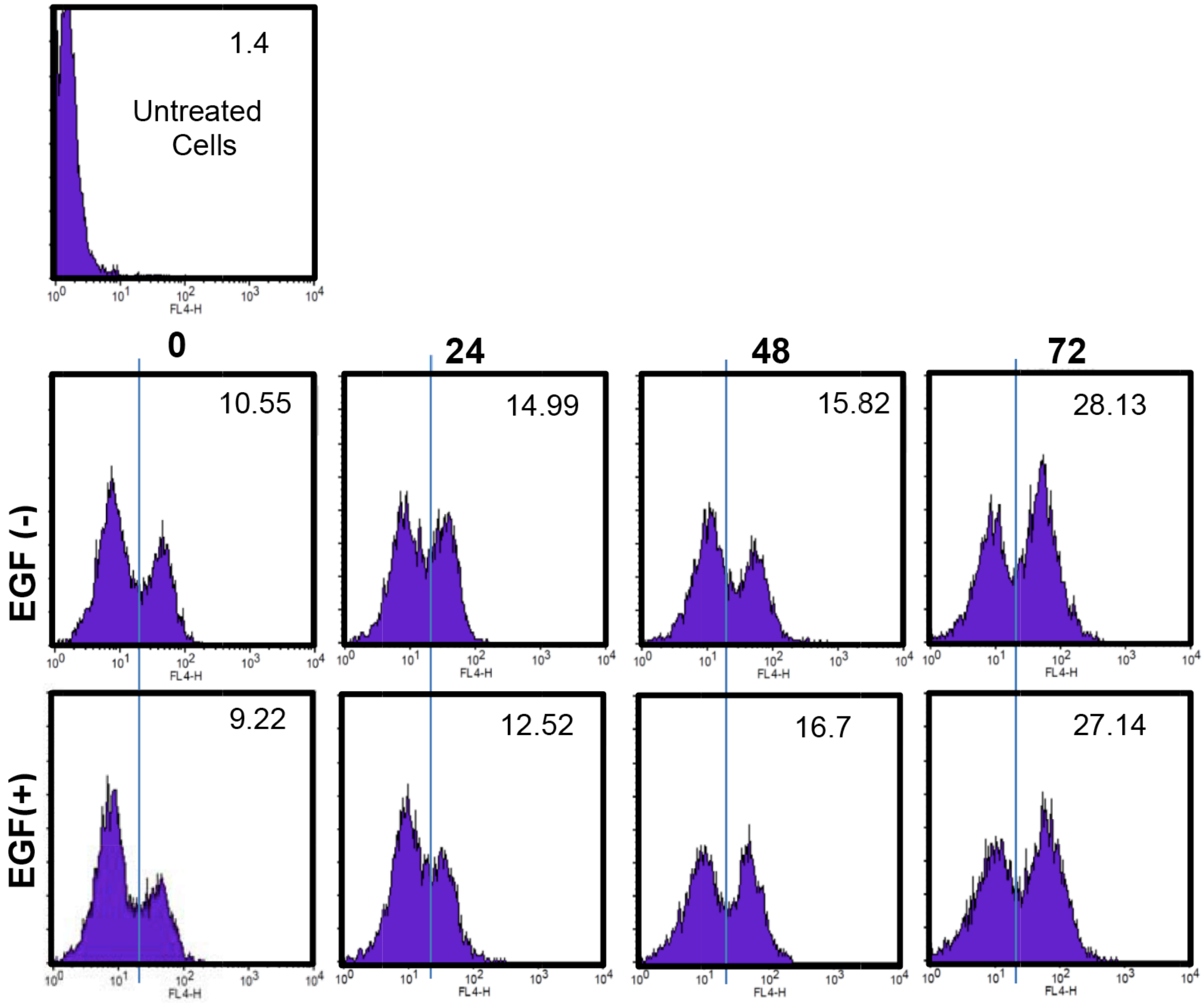

Supplementary Figure S5: Intracellular ROS levels in p22phox KD cells. A total of 5×10^5 cells/well was incubated with 100 ng/mL hrEGF for 0, 24, 48, or 72 h. The percentage of cells in the upper section of the histogram indicates normal cells with little or no fluorescence (mean value: 1.47), whereas the lower side indicates cells with higher fluorescence, representing increased intracellular ROS generation over time.

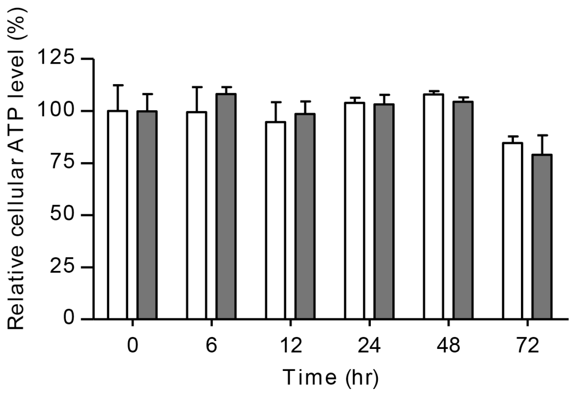

Supplementary Figure S6: Intracellular ATP levels were not altered in EGF-treated A549 cells. A total of 5×10^4 cells/well was incubated with 100 ng/mL hrEGF for 0, 6, 12, 24, 48, or 72 h, and intracellular ATP was measured using a luminometer (VICTOR3 Multilabel Reader; PerkinElmer) with an ENLITEN ATP assay kit. Relative intracellular ATP levels are shown in the bar graph. Data represent the mean ± SD for each group ($n = 6$). *$P < 0.05$ for the control group (white bar) versus the EGF-treated group (black bar) by Student's *t*-tests.

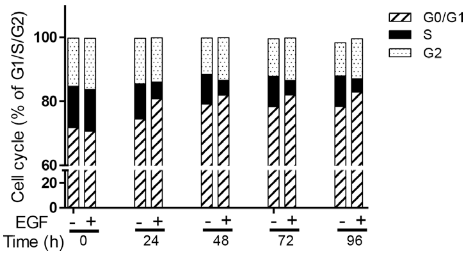

Supplementary Figure S7: Cell cycle of A549 cells. A549 cells were incubated with 100 ng/mL hrEGF for 0, 24, 48, 72, or 96 h. The changes in cell cycle viabilities after treatment with EGF were then measured and analyzed using PI staining with flow cytometry. Representative results of three independent experiments are shown.

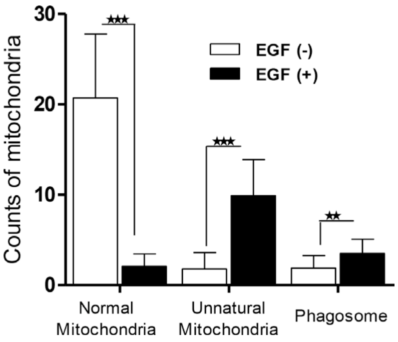

Supplementary Figure S8: Quantification of mitochondria and phagosomes of tumor tissue in electron microscopic images. EGF-treated cells exhibited some structural changes in control tumor tissue and tumor tissues from mice injected with 1 mg/kg EGF, including swelling and differences in mitochondrial morphology, as revealed by electron microscopy (EM; original magnification, 12000×). Normal mitochondria, dysfunctional mitochondria, and phagosomes were enumerated in 10 EM images. Data were analyzed by unpaired *t*-tests. $^{**}p < 0.01$, $^{***}p < 0.001$.

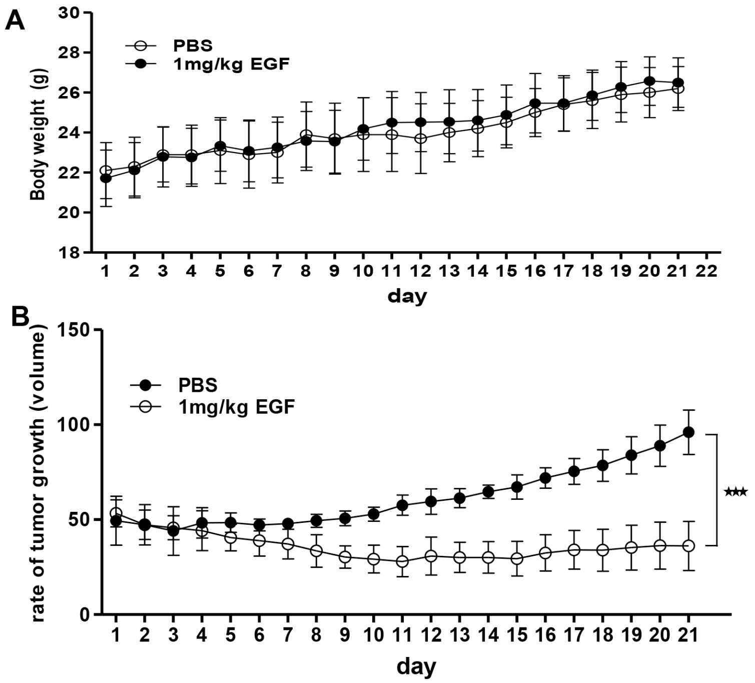

Supplementary Figure S9: Body weight and tumor size measurement in the lung cancer animal model. (**A**) Body weights of tumor-bearing control mice (black circles) and EGF-injected tumor-bearing mice (white circles). The x-axis values represent the number of days after EGF injection. (**B**) Tumor size was calculated by multiplying the shortest and longest diameters using digital calipers. Treatments with EGF were administered after a couple of weeks when tumors reached an average volume of 50 mm3. Tumor tissues were derived from 6-week-old immunodeficient mice (nu/nu) that had been treated daily with EGF (0 or 1 mg/kg/PBS) for 21 days. Differences between PBS-injected tumor-bearing control mice (n = 6, whit circles) and EGF-injected tumor-bearing mice (n = 8, black circles) were analyzed using unpaired *t*-tests. $^{***}p < 0.001$. Data are means and standard deviations.

MATERIALS AND METHODS

EGFR1, $p22^{phox}$, or Ref-1 knockdown (KD) and U6-GFP-control stable A549 cell lines (EGFR1 KD, $p22^{phox}$ KD, Ref-1 KD, and control KD cells, respectively)

Bacterial glycerol stocks of pLKO.1-puro-human EGFR1 (ErBb1), -human $p22^{phox}$, human Ref-1, and -U6-EGFP of lentiviral plasmid vectors were purchased from Sigma Aldrich (St. Louis, MO, USA). The vectors were cotransfected into 293FT cells (Invitrogen, Carlsbad, CA, USA) with plasmids of lentiviral third-generation-coating proteins (pMDLG/pRRE, pRSV-REV, and pMD.G) for 36–49 h. The viral particles were then concentrated from 293FT host cells using a 0.45-µm syringe filter and Lenti-X concentrator (Clontech, Mountain View, CA, USA). A549 cells were infected with these particles using 8 µg/mL polybrene (hexadimethrine bromide; H9268; lot #SLBN2602V; Sigma Aldrich) in order to generate stable knockdown cell lines for the indicated specific target genes (*EGFR1*, *p22*phox, and *Ref-1*). After four passages of A549 cell selection using puromycin (BML-GR312; lot #03261415; Enzo Life Sciences, Farmingdale, NY, USA), each stable cell line with knockdown of a specific target gene was verified with RT-PCR and western blotting.

Authentication of NSCLCs and knockdown of specific genes in A549 cell lines by analysis of short tandem repeats (STRs)

Authentication of the cells (A549, EKVX, NCI-H23, NCI-H266, NCI-H322M, and NCI-H522; Supplementary Table S1) was performed by analysis of STRs and comparison with the original cell line (Supplementary Table S2). To demonstrate the derivation of control KD, EGFR1 KD, $p22^{phox}$ KD, and Ref-1 KD cell lines from the parental A549 cell line, STRs of all five cell lines were performed and compared with the original parental A549 cells. The cells exhibited consistency with parental A549 cells (Supplementary Table S3). All cell-specific tetranucleotide repeat loci and the amelogenin gender-determining marker were amplified using AmpFLSTR Identifiler Plus PCR Amplification Kit (Applied Biosystems, CA, USA) and analyzed using a 3730 DNA analyzer and peak scanner (Applied Biosystems).

Colony formation assay

Untreated-control and pretreated-EGF cells were plated at a low density (300 ~ 600 cells/well) in RPMI-1640 media. Cells were fed fresh medium in the presence of EGF (100 ng/ml) once every three days. After two weeks of culture, cells were fixed with 4% formaldehyde for 10 min and stained with crystal violet (0.05%, 2 h).

Measurement of mRNA expression levels

Total RNA was extracted from A549 cells using TRIzol reagent (Invitrogen), and 1 µg total RNA from each sample was reverse transcribed. The RT-PCR protocol was as follows: 27–30 cycles at 94°C for 30 s, 58°C for 50 s, and 72°C for 1 min. The primer sequences for human *EGFR1*, human *EGFR2*, and human *Ref-1* are shown in Supplementary Table S2). As an internal standard, human *GAPDH* mRNA was amplified and analyzed under identical conditions using a pair of specific primers.

Analysis of western blotting

Western blotting was performed using commercial antibodies against EGFR1 (1:1000 dilution; cat. #2232; Cell Signaling Technology) and β-actin (N-21; 1:5000 dilution; cat. #sc-1616; Santa Cruz Biotechnology, Santa Cruz, CA, USA).

Detection of intracellular ROS generation

For the flow cytometric analysis of ROS production, EGF-treated cells were monitored by 2′,7′-dichlorofluorescin diacetate (H2DCFDA; CellRox Deep Red; cat. #C10422; Molecular Probes Inc., OR, USA). Briefly, control and treated cells were loaded with 5 µM H2DCFDA in RPMI 1640 medium for 30 min at 37°C. The cells were then washed twice with fresh DPBS. Data were obtained using a flow cytometer (FACSCalibur; BD Bioscience, CA, USA) with an FL-4 filter and analyzed using Cell Quest Pro software (BD Bioscience).

ATP measurement

Intracellular ATP was measured using an ENLITEN ATP assay system bioluminescence detection kit (#TB267; Promega Corp., Madison, WI, USA), according to the manufacturer's instructions. Briefly, 5×10^4 cells/well were incubated with EGF for different times. Supernatants were then removed, and cells were washed with PBS. The cells of each well were lysed, and intracellular ATP was quantified using a fluorometer (VICTOR3 Multilabel Reader; PerkinElmer).

Cell cycle analysis

A549, control KD, $p22^{phox}$KD, or Ref-1KD was treated with 100 ng/ml EGF. The cell cycle was analyzed using a flow cytometer.

Supplementary Table S1: List of the human non-small cell lung cancer (NSCLC) cell lines used in this study

No.	Name	Tissue	Type of tumor	Morphology	Provenance
1	A549	Lung	Carcinoma, NSCLC	Epithelial	Caucasian 59year, male
2	EKVX	Lung	Adenocarcinoma, NSCLC		Xenograft nude mouse
3	NCI-H23	Lung	Adenocarcinoma, NSCLC	Epithelial	Ethnicity black, 51year, male
4	NCI-H226	Lung	Squamous cell carcinoma Mesothelioma , NSCLC	Epithelial	Derived from metastatic site
5	NCI-H322M	Lung	Bronchi Alveolar carcinoma, NSCLC		Athymic nude
6	NCI-H522	Lung	Adenocarcinoma, NSCLC	Epithelial	Caucasian 58year, male

Supplementary Table S2: Comparison of the short tandem repeat profiles used for NSCLC cell line authentication (P: existing STR information of original parental cells versus U: used cells)

	A549		EKVX		NCI-H23		NCI-H266		NCI-H322M		NCI-H522	
	P	U	P	U	P	U	P	U	P	U	P	U
Amelogenin	X, Y	X, Y	X, Y	X, Y	X	X	X, Y	X, X	X, Y	X, Y	X	X
CSF1PO	10, 12	10, 12	9	9	10	10	10, 11	10, 11	12	12	10	10
D13S317	11	11	11	11	12	12	13, 14	13, 14	12	12	10	10
D16S539	11, 12	11, 12	9, 11	9, 11	11	11	9, 12	9, 9	11, 12	11, 12	12	12
D5S818	11	11	11, 12	11, 12	12, 13	12, 13	11, 12	11, 12	11	11	11	11
D7S820	8,11	8,11	9, 12	9, 12	9, 10	9, 10	8, 10	8, 10	11	11	9, 10	9, 10
TH01	8, 9.3	8, 9.3	9.3	9.3	6	6	8, 9.3	8, 9.3	7	7	7, 9.3	7, 9.3
TPOX	8, 11	8, 11	8, 11	8, 11	8, 9	8, 9	8, 11	8, 11	8	8	9, 10	9, 10
vWA	14	14	15	15	16, 17	16, 17	17	17	17	17	17	17
% Match P vs. U	100 %		100 %		100 %		94.12 %		100 %		100 %	

Supplementary Table S3: Comparison of the short tandem repeat (STR) profiles of A549 cells for A549 cell line authentication (parental A549 cells versus A549 cells with specific gene knockdown)

	Parental A549	Control KD of A549	$P22^{phox}$ KD of A549	Ref-1 KD of A549
D8S1179	13, 14	13, 14	13, 14	13, 14
D21S11	29	29	29	29
D7S820	18, 11	18, 11	18, 11	18, 11
CSF1PO	10, 12	10, 12	10, 12	10, 12
D3S1358	16	16	16	16
TH01	8,11	8,11	8,11	8,11
D13S317	8, 9.3	8, 9.3	8, 9.3	8, 9.3
D16S539	11, 12	11, 12	11, 12	11, 12
D2S1338	23, 24	23, 24	23, 24	23, 24
D19S433	13	13	13	13
vWA	14	14	14	14
TPOX	8, 11	8, 11	8, 11	8, 11
D18S51	14, 17	14, 17	14, 17	14, 17
Amelogenin	X, Y	X, Y	X, Y	X, Y
D5S818	11	11	11	11
FGA	23	23	23	23

Supplementary Table S4: RT-PCR primer sequences

Gene	5¢ sequence	3¢ sequence	bp	GenBank Accession Number
P2Y	CGTCTCCTCGTCGTTCAAAT	TCAGCACGTACAAGAAGTCG	203	NM_002563.4
EGFR1	TGTGATCCAAGCTGTCCCA	GCCAAGCCTGAATCAGCAAA	664	NM_005228.3
EGFR2	TGCTCATCGCTCACAACCAA	AATGCCAGGCTCCCAAAGAT	877	NM_004448.3
$P22^{PHOX}$	TGGTACTTTGGTGCCTACTC	GCGGTCATGTACTTCTGT	119	NM_000101.3
REF-1	AAACCTCACCCAGTGGCAAA	AATTCAGCCACAATCACCCG	242	S43127.1
EGR-1	CTTCAGTCGTAGTGACCACCT	ATGTCTGAAAGACCAGTTGAG	160	NM_001964.2
PTEN	CGAACTGGTGTAATGATATGT	CATGAACTTGTCTTCCCGT	330	NM_000314.6
GAPDH	GACCCCTTCATTGACCTC	GCTAAGCAGTTGGTGGTG	370	BC083511.1
